# Artificial Intelligence in Veterinary Neurology: Comparative Insights From Human Medicine and Cross‐Species Technology Transfer

**DOI:** 10.1002/vms3.71152

**Published:** 2026-08-10

**Authors:** Kianoush Saberi, Rosull Saadoon Abbood, Sarah F. Al‐Taie, Aseel Smerat, Abdullaev Makhmudjon Mukhamedovich, Maksudova Malika Khamdamjonovna, Mohammad Hedayatinia, Arash Zarei, Mehrdad Nourizadeh, Amir Arsalan Ghahari, Mehrdad Neshat Gharamaleki, Fatemeh Malekinejad, Reza Akhavan‐Sigari

**Affiliations:** ^1^ Department of Anesthesiology, Medical Faculty Imam Khomeini Hospital Complex, Tehran University of Medical Sciences Tehran Iran; ^2^ Medical Laboratory Techniques Department, College of Health and Medical Technology University of Al‐Maarif Ramadi, Anbar Iraq; ^3^ College of Science, Department of Biotechnology University of Baghdad Baghdad Iraq; ^4^ Hourani Center for Applied Scientific Research Al‐Ahliyya Amman University Amman Jordan; ^5^ Department of Mechatronics and Robotics, Faculty of Electronics and Automation Tashkent State Technical University Named After Islam Karimov Tashkent Uzbekistan; ^6^ Department of Faculty and Hospital Therapy No. 2, Nephrology and Hemodialysis Tashkent State Medical University Tashkent Uzbekistan; ^7^ Department of Veterinary Medicine, Sho.C. Islamic Azad University Shoushtar Iran; ^8^ Department of Veterinary Medicine, TaMS.C. Islamic Azad University Tabriz Iran; ^9^ Department of Clinical Sciences, TaMS.C Islamic Azad University Tabriz Iran; ^10^ Student Research Committee Tabriz University of Medical Sciences Tabriz Iran; ^11^ ATOS Dr. Schneiderhan GmbH and Isar Klinikum Munich Germany; ^12^ Department of Health Care Management and Clinical Research Collegium Humanum Warsaw Management University Warsaw Poland

**Keywords:** artificial intelligence, comparative neurology, cross‐species technology transfer, deep learning, machine learning, one health, veterinary neurology

## Abstract

**Background:**

Artificial intelligence (AI) is increasingly explored in veterinary neurology for pattern recognition, prediction and clinical decision support, with relevance to comparative and translational neuroscience.

**Objectives:**

This review examines AI applications in veterinary neurology, compares them with human neurology and evaluates cross‐species technology transfer within a One Health framework.

**Methods:**

A narrative review synthesized evidence across AI domains in veterinary neurology, including neuroimaging and radiomics, electrophysiology and seizure detection or forecasting, gait and pain assessment, morphometric biomarker discovery, prognostic modelling and laboratory diagnostic tools. Human studies were considered to identify translational opportunities, methodological challenges and the value of transfer learning and domain adaptation.

**Results:**

Available evidence suggests that AI holds promise for pattern recognition, prediction and decision support in veterinary neurology. Reported applications include canine brain tumour classification, spinal lesion grading, seizure monitoring and quantitative gait analysis, with encouraging performance. However, most applications remain proof‐of‐concept. The evidence base is dominated by retrospective single‐centre studies with small samples, heterogeneous protocols and limited prospective or external validation. Model calibration, uncertainty reporting and clinically relevant error trade‐offs are often insufficiently addressed. Transfer learning and domain adaptation may help overcome limited veterinary datasets, while naturally occurring neurological disease in dogs may also support refinement of human AI systems.

**Conclusions:**

AI in veterinary neurology is a promising but early field. Future progress will require multi‐centre collaboration, standardized data practices, explainable and ethically governed models and stronger One Health partnerships to support safe translation.

## Graphical abstract

Overview of major artificial intelligence applications in veterinary neurology, including neuroimaging, seizure prediction, gait and pain assessment, and prognostic modeling. The figure also highlights cross‐species technology transfer and key challenges, such as limited data, lack of standardization, and ethical concerns.

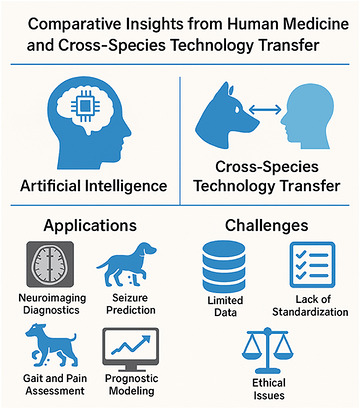

## 1 Introduction

Artificial intelligence (AI) has moved from a conceptual idea in computer science to a set of practical tools used across medical diagnostics, prognostics and clinical decision support (Khosravi et al. 2024). In human neurology, AI systems have demonstrated measurable benefits in specific settings, particularly in neuroimaging triage and segmentation, electroencephalography (EEG) analysis and wearable sensor‐based monitoring, although real‐world impact varies by task, data quality and level of clinical integration. These approaches, spanning machine learning (ML) and deep learning (DL), can learn complex patterns from high‐dimensional data and may support faster or more consistent interpretation when validated and deployed appropriately (Esteva et al. 2021; J. Zhang et al. 2022; Rizzo 2025). AI applications in veterinary neurology are an emerging area that is advancing rapidly, in part because companion animals such as dogs and cats develop naturally occurring neurological disorders with clinically relevant parallels to human disease, including gliomas, idiopathic epilepsy, spinal cord injury and Chiari‐like malformation (CM) (Appleby and Basran 2022; Akinsulie et al. 2024). This biological and clinical overlap creates opportunities for cross‐species technology transfer, where model architectures, training strategies and representation learning developed in human medicine can be adapted to veterinary contexts. Importantly, translation is potentially bidirectional: Long‐term, real‐world monitoring in canine epilepsy can contribute to the development and robustness testing of seizure detection and forecasting methods; canine gliomas can serve as large‐animal neuro‐oncology imaging models; and quadrupedal gait analysis can broaden biomechanical variability that may improve generalizability of movement analytics (Akbarein et al. 2025; Albadrani et al. 2024). This reciprocal perspective aligns with One Health, which emphasizes interconnected human, animal and environmental health and encourages shared infrastructure and governance for data‐driven innovation (Appleby and Basran 2022; Akinsulie et al. 2024). However, several barriers limit routine clinical adoption of veterinary neurology AI. Available datasets are often small, heterogeneous and inconsistently standardized across diagnostic equipment, imaging protocols, breeds and institutions (Pomerantz et al. 2023; Burti et al. 2024). External validation and prospective evaluation are uncommon, and reporting of clinically relevant metrics such as calibration, uncertainty and error trade‐offs is frequently incomplete (Gulzar and Hussain 2023; Basran and Appleby 2024). In addition, veterinary‐specific regulatory pathways for neurology‐focused AI remain limited, and ethical considerations are especially salient for prognostic tools that could influence irreversible decisions, including surgical escalation or euthanasia (Coghlan and Quinn 2024).

Against this background, this narrative review synthesizes AI applications relevant to veterinary neurology within a comparative framework. We summarize advances in neuroimaging and radiomics, electrophysiology and seizure detection or forecasting, gait and pain assessment, morphometric biomarker discovery, prognostic modelling and educational or laboratory tools. Throughout, we emphasize methodological limitations that affect translation, highlight evidence gaps and sources of heterogeneity and outline practical and ethical requirements for moving from proof‐of‐concept performance towards clinically trustworthy deployment.

## 2 Methods

This narrative review was designed to synthesize current evidence on AI applications relevant to veterinary neurology and to compare these developments with analogous advances in human neurology within a One Health framework. Literature searches were performed in PubMed, Scopus, Web of Science and Google Scholar to identify primarily peer‐reviewed articles published between January 2010 and January 2026. Search terms included combinations of ‘artificial intelligence’, ‘machine learning’, ‘deep learning’, ‘veterinary neurology’, ‘canine epilepsy’, ‘seizure detection’, ‘seizure forecasting’, ‘veterinary MRI’, ‘radiomics’, ‘neuroimaging’, ‘gait analysis’, ‘pain recognition’, ‘prognostic modeling’, ‘clinical decision support’, ‘cross‐species translation’ and ‘One Health’. Additional relevant sources were identified by screening the reference lists of key original studies, reviews and position or guidance papers.

Studies were considered eligible if they addressed veterinary neurology, neurodiagnostic or neuromonitoring workflows, AI/ML‐based analytical methods, or human neurology applications with clear translational relevance to veterinary medicine. Priority was given to peer‐reviewed original studies that reported the clinical task, model input data, AI/ML method, validation approach and performance outcomes. Review articles, methodological papers and professional guidance documents were included when they provided important context regarding validation, explainability, regulation, ethics or implementation. Sources were excluded when they were unrelated to neurological disease or neurodiagnostic workflows, lacked sufficient methodological detail to interpret performance claims or made broad statements about AI without domain‐specific evidence.

Because this article is a narrative review rather than a systematic review, no formal PRISMA‐based screening workflow, meta‐analysis or risk‐of‐bias scoring was performed. The synthesis may therefore be affected by publication bias, selective availability of veterinary AI studies and heterogeneity in reporting standards across studies. To reduce overinterpretation, the evidence is discussed according to validation status, clinical maturity, methodological limitations and translational relevance rather than headline performance metrics alone.

## 3 AI Foundations and Cross‐Disciplinary Transfer

AI refers to computational systems designed to perform tasks that typically require human cognition, including pattern recognition, classification and decision support (Da Mota Gomes 2025). Within AI, ML improves performance by learning from data, while DL uses multi‐layer neural networks to learn hierarchical representations directly from high‐dimensional inputs such as MRI, EEG and motion data (Da Mota Gomes 2025; Hespel et al. 2022). In neurology, these methods can support more consistent interpretation and triage for specific tasks when they are trained, validated and clinically integrated appropriately. However, performance is strongly shaped by data quality, acquisition protocols, cohort composition and workflow design (Esteva et al. 2021; J. Zhang et al. 2022; Rizzo 2025).

Neurology‐focused AI commonly relies on supervised learning for labelled prediction tasks, such as classifying MRI lesions, and may also use unsupervised, semi‐supervised and self‐supervised approaches to leverage partially labelled or unlabelled data. This is particularly relevant in veterinary settings, not because expert annotations are entirely absent, but because existing annotations are often embedded in heterogeneous diagnostic reports, imaging interpretations, cytology or histology records and clinician case summaries. These data are frequently non‐standardized, incompletely structured and siloed across institutions, making them difficult to clean, harmonize and use as training data for machine‐learning pipelines (Pereira et al. 2023; Hennessey et al. 2022). Importantly, quantitative approaches such as radiomics should be framed as complementary to qualitative clinical interpretation rather than as replacements, because appropriate use still depends on domain expertise, clinical context and imaging quality control (Monsour et al. 2022).

Transfer learning is central to veterinary neurology because it enables models pretrained on large human datasets to be adapted to veterinary tasks such as canine brain tumour classification and spinal lesion grading, reducing the need for large veterinary‐specific cohorts (Banzato et al. 2018; Lee et al. 2024). However, the benefits of transfer are not automatic. Cross‐site and cross‐species dataset shift, class imbalance and limited sample sizes can increase overfitting risk and yield optimistic performance estimates when studies rely only on internal validation. Where feasible, reporting should clarify the validation design, include external test sets and discuss clinically meaningful properties such as calibration and uncertainty, particularly for decision‐support use cases (Gulzar and Hussain 2023; Basran and Appleby 2024). Translation can also be bidirectional. Naturally occurring neurological disease in dogs, including idiopathic epilepsy, provides ecologically valid data streams, and canine intracranial EEG (iEEG) has been used to evaluate seizure forecasting algorithms under long‐term, real‐world conditions that are difficult to replicate in human studies (Packer et al. 2018; Nejedly et al. 2019). Beyond epilepsy, veterinary neurology can also contribute to human AI by providing broader morphological and biomechanical diversity for movement analytics, real‐world outcomes after spinal cord injury and rehabilitation and pain or function monitoring signals that help evaluate generalizability under heterogeneous conditions.

Within a One Health framework, cross‐species AI transfer is most effective when it is operationalized through shared standards, transparent validation and explicit domain adaptation to account for differences in anatomy, physiology, acquisition protocols and clinical endpoints. In practical terms, such transfer is most defensible when the target task and clinical endpoint are meaningfully comparable across species, performance is validated in the receiving species rather than assumed from human data alone and the anticipated benefit includes clear veterinary as well as translational value. For example, although some EEG dynamics are conserved across species, skull geometry and electrode placement can alter signal characteristics and may require tailored preprocessing and harmonization (Lustgarten et al. 2020; Hu and Zhang 2020). Similarly, cross‐species comparisons should avoid assuming that apparently analogous clinical outcomes have identical functional or welfare meaning across humans and animals. Species‐specific endpoints, owner‐reported quality of life and clinician‐assessed function may need to be incorporated when veterinary data are used for translational AI development. Aligning cross‐species efforts around harmonized metadata, standardized endpoints and transparent validation can improve interpretability and support more meaningful cross‐cohort comparison. Overall, veterinary neurology can accelerate AI development by adapting mature human methods while contributing complementary disease models and diverse datasets that may improve robustness and generalizability across settings (Appleby and Basran 2022; Mardones et al. 2017; Bowsher et al. 2021). These gains are most defensible when methodological limitations are stated explicitly and conclusions are aligned with the strength of available evidence (Gulzar and Hussain 2023; Basran and Appleby 2024).

## 4 AI in Neuroimaging and Imaging Biomarker Discovery

**FIGURE 1:**
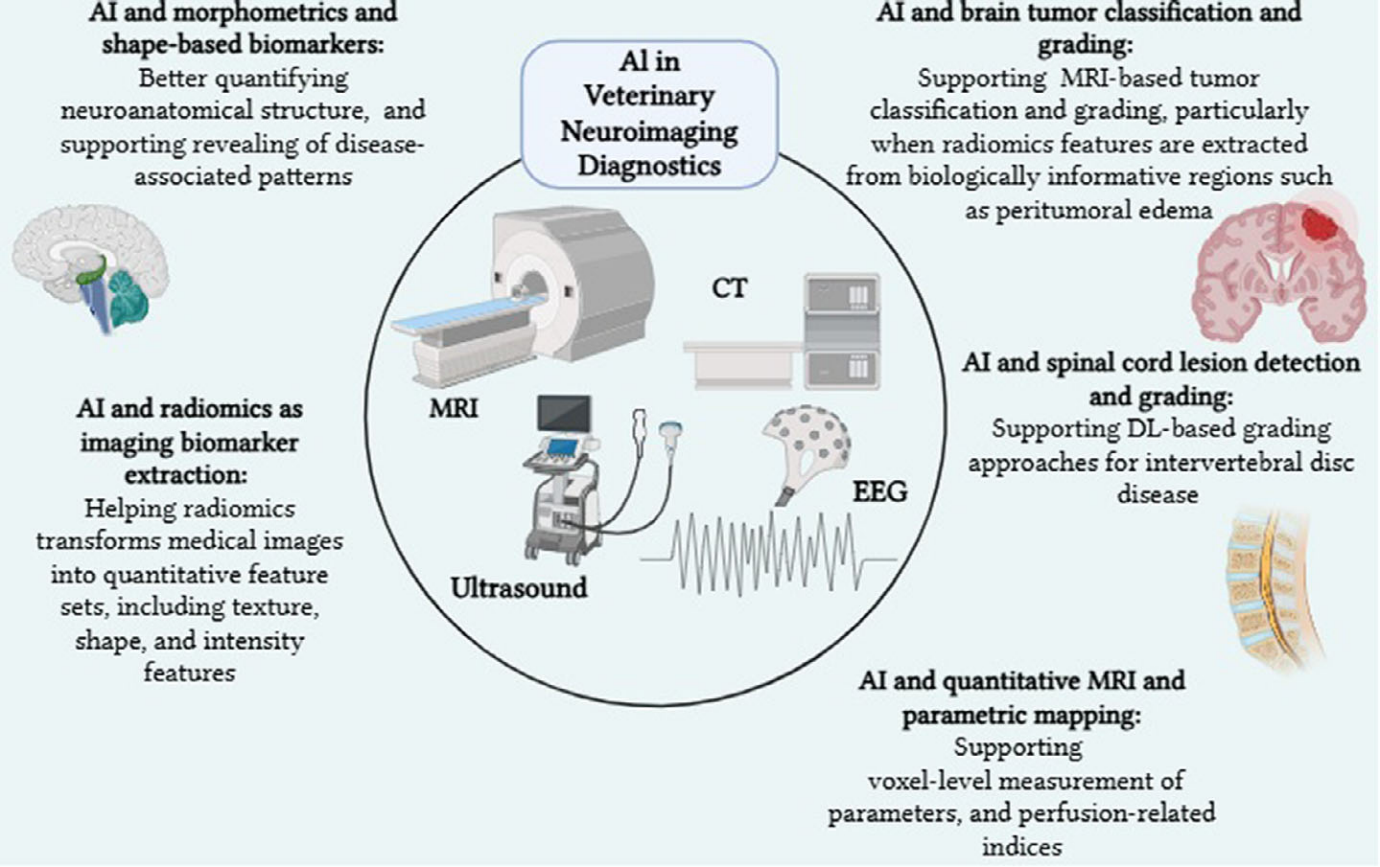
Representative artificial intelligence applications in veterinary neuroimaging diagnostics. The schematic highlights major research directions, including MRI‐based brain tumour classification and grading, spinal cord lesion detection and grading, quantitative MRI and parametric mapping, radiomics‐based biomarker extraction and morphometric or shape‐based biomarker discovery. These applications illustrate the technical feasibility of AI‐assisted neuroimaging in veterinary neurology, but most remain research‐stage or decision‐support candidate tools. Clinical translation will require standardized imaging protocols, reproducible analysis pipelines, external validation, uncertainty reporting and veterinarian‐in‐the‐loop interpretation.

Neuroimaging is central to both human and veterinary neurology for lesion localization, disease characterization and treatment planning. In parallel with human neuroradiology, veterinary neuroimaging research has increasingly shifted from purely qualitative visual interpretation towards quantitative image analysis supported by AI, including DL and radiomics workflows (Esteva et al. 2021; Zhang et al. 2022; Monsour et al. 2022). In human neurology, several AI‐enabled neuroimaging applications have progressed to regulated clinical use in specific tasks such as stroke triage and lesion segmentation, which provides a useful reference point for what is required for translation. In veterinary neurology, most imaging‐focused AI remains at a research or proof‐of‐concept stage, but the literature indicates clear technical feasibility across several clinically relevant problems (Figure 1).

### 4.1 Diagnostic Classification and Grading Tasks

#### 4.1.1 Brain Tumour Classification and Grading

Studies in canine neuro‐oncology suggest that AI can support MRI‐based tumour classification and grading, particularly when radiomics features are extracted from biologically informative regions such as peritumoural oedema (Banzato et al. 2018; Barge et al. 2023). For example, Banzato et al. (2018) used transfer learning with a GoogLeNet‐based convolutional neural network (CNN) to differentiate canine meningiomas from gliomas, reporting accuracy above 91%. Barge et al. (2023) applied a radiomics pipeline that segmented enhancing, non‐enhancing and peritumoural oedema regions, extracted high‐dimensional texture features and trained a support vector machine model, reporting high accuracy for tumour subtype and grade prediction. Beyond subtype discrimination, a clinically important veterinary application is the differentiation of neoplastic from inflammatory intra‐axial brain disease, because these entities can show overlapping MRI characteristics and histopathologic confirmation is often not feasible in routine practice. In a retrospective study of 119 dogs, MRI texture analysis combined with a random forest classifier achieved 85% accuracy in the test set for distinguishing glial neoplasia from non‐infectious inflammatory meningoencephalitis, supporting the potential value of quantitative imaging for broad etiologic classification when visual interpretation is equivocal (Wanamaker et al. 2021). However, the same study showed more limited performance for glioma grading and inflammatory subtype classification, underscoring the current gap between promising discrimination of broad disease categories and finer‐grained diagnostic stratification. These findings are broadly consistent with human glioma radiomics literature, where peritumoural texture has also been associated with improved discrimination of tumour biology, although veterinary evidence remains limited by cohort size, centre‐specific acquisition and incomplete external validation (Wanamaker et al. 2021; Tan et al. 2024).

#### 4.1.2 Spinal Cord Lesion Detection and Grading

In spinal imaging, DL‐based grading approaches have been explored for canine intervertebral disc disease (IVDD). Niemeyer et al. (2024) trained a VGG‐based CNN on sagittal T2‐weighted MRI to estimate lesion severity and reported high overall accuracy, although performance declined in rare or atypical cases, highlighting sensitivity to class imbalance and distribution shift (Lee et al. 2024; Niemeyer et al. 2024). Translational interpretation of spinal outcome data requires particular caution because clinically acceptable outcomes may differ substantially between species. Dogs and cats may retain acceptable quality of life despite marked imaging abnormalities, whereas less dramatic pathology in humans may be associated with severe functional disability. Therefore, spinal AI models should align imaging endpoints with species‐specific functional, welfare and owner‐reported outcomes rather than assuming direct equivalence with human disability metrics.

This mirrors limitations seen in human spinal imaging AI and reinforces the need for harmonized acquisition, robust validation and careful handling of minority classes before clinical use (Table 1). It also highlights the importance of veterinarian‐in‐the‐loop deployment, in which AI outputs are interpreted as decision‐support signals under qualified clinical supervision rather than as autonomous diagnostic conclusions. This approach is consistent with recent ACVR/ECVDI guidance emphasizing transparency, validation, patient safety and the continued responsibility of veterinary professionals when AI is used in diagnostic imaging (Appleby et al. 2025).

**TABLE 1:** Representative AI studies in veterinary neurology: diagnostic and prognostic applications, validation context and translational read.

Study (year)	Species	Clinical task	AI method	Sample size	Validation type	Main finding	Main limitation	Clinical readiness	Reference
Banzato et al. (2018)	Dog	MRI classification of intracranial neoplasia (meningioma vs. glioma)	GoogLeNet CNN with transfer learning	120 MRI cases	Internal validation	91%–94% classification accuracy	Single‐species, limited external validation	Experimental/proof‐of‐concept	(Banzato et al. 2018)
Barge et al. (2023)	Dog	Glioma subtype and grade prediction from MRI radiomics	SVM with radiomic features	50 tumour cases	Internal validation	Up to 94% subtype accuracy; 87% grade accuracy	Small cohort, protocol sensitivity, no clear external validation	Experimental/proof‐of‐concept	(Barge et al. 2023)
Flegel et al. (2024)	Dog	Etiology classification in epilepsy (idiopathic vs. structural)	Bayesian networks; random forest	328 seizure cases	Internal validation	96.9% overall accuracy; 100% specificity for structural epilepsy	Retrospective design, clinical substitution risk without external validation	Decision‐support candidate, not stand‐alone	(Flegel et al. 2024)
Low et al. (2025)	Dog	Recovery prediction after thoracolumbar disc extrusion surgery	XGBoost	162 surgical cases	Internal validation	AUC 0.95; 89% accuracy for ambulation prediction	Needs external and prospective validation	Prognostic support candidate	(Low et al. 2025)
Spiteri et al. (2019)	Dog	Morphometric prediction of Chiari‐like malformation pain/syringomyelia	Shape metrics with random forest	45 CKCS dogs	Internal validation	AUC about 0.82; 93% sensitivity for syrinx prediction	Small breed‐specific cohort	Experimental biomarker tool	(Spiteri et al. 2019)
Wanamaker et al. (2021)	Dog	Differentiation of neoplastic versus inflammatory intra‐axial brain disease	MRI texture analysis + random forest	119 dogs	Train/test split; internal test set	85% test accuracy; 89% sensitivity; 81% specificity	Single‐centre, retrospective, manual 2D ROI, non‐standardized acquisition	Experimental/proof‐of‐concept	(Wanamaker et al. 2021)

### 4.2 Quantitative MRI (qMRI) and Parametric Mapping

qMRI enables voxel‐level measurement of parameters such as apparent diffusion coefficient (ADC), fractional anisotropy (FA) and perfusion‐related indices, and may improve objectivity compared with purely qualitative assessment (Li et al. 2023). However, its application in veterinary neurology remains constrained by small datasets, heterogeneous acquisition protocols and limited cross‐site standardization. Spatial resolution is an additional challenge in dogs and cats because their smaller brain volumes mean that routine clinical voxel sizes may encompass proportionally more heterogeneous tissue, increasing spatial averaging and potentially reducing the stability of quantitative measures.

Céré et al. (2024) emphasized that clinical translation is limited by the lack of standardized acquisition and analysis pipelines in dogs and cats, a challenge that parallels the early development of human qMRI before broader harmonization efforts were established. Diffusion‐weighted MRI studies in canine epilepsy support this cautious interpretation. In a prospective study of dogs with idiopathic epilepsy, interictal ADC mapping identified region‐specific diffusion differences, particularly in the piriform lobe, including the amygdala, and in the semioval centre, suggesting that quantitative diffusion imaging may detect abnormalities not visible on routine structural MRI (Hartmann et al. 2017). However, the study was based on a small cohort, manual ROI placement and a single imaging setup. Therefore, current veterinary qMRI findings should be interpreted as preliminary imaging biomarker signals rather than clinically validated decision‐support tools.

### 4.3 Morphometrics and Shape‐Based Biomarkers

Morphometric analysis quantifies neuroanatomical structure and can reveal disease‐associated patterns that may be subtle or difficult to capture with manual measurements (Rebsamen et al. 2020; Rahmani et al. 2023). In veterinary neurology, automated morphometrics has been most visible in CM and syringomyelia (SM) research in Cavalier King Charles Spaniels. Spiteri et al. (2019) used deformation mapping and ML to identify morphological signatures in the caudal cranial fossa and atlanto‐occipital region associated with CM‐related pain and SM, reporting moderate‐to‐good discriminative performance.

These findings align conceptually with human Chiari malformation studies, where posterior fossa morphometry and crowding at the foramen magnum are clinically meaningful (Yan et al. 2016; Saygı et al. 2024). However, cross‐species interpretation should remain cautious because similar anatomical measurements may not map directly onto equivalent clinical disability, pain or quality‐of‐life outcomes across humans, dogs and cats. Beyond CM/SM, volumetric and regional atrophy mapping is well established in human neurodegeneration research and is beginning to appear in veterinary contexts, including canine cognitive dysfunction and epilepsy‐associated hippocampal or temporal lobe volumetry (Jasodanand et al. 2025; Akan et al. 2025). These applications may provide objective endpoints for monitoring and trials, but they remain sensitive to segmentation quality, diagnostic equipment, imaging protocol variability and cohort representativeness.

### 4.4 Radiomics as Imaging Biomarker Extraction

Radiomics transforms medical images into quantitative feature sets, including texture, shape and intensity features, that can be linked to histology, phenotype or prognosis (Zwanenburg et al. 2020). In canine gliomas, radiomic features derived from non‐enhancing tumour core and peritumoural oedema have shown utility for predicting histologic type and grade using classical ML models such as support vector machines or random forests (Spiteri et al. 2019). This is conceptually consistent with human glioma radiomics research, where imaging signatures have been associated with molecular and clinical endpoints (Gao et al. 2020; Chaddad et al. 2025).

To avoid overstating clinical readiness, veterinary radiomics should currently be framed as a promising biomarker discovery approach rather than a validated diagnostic substitute. Its translation will require standardized segmentation, reproducible feature extraction, multi‐centre testing and reporting of model performance under realistic variation in diagnostic equipment and imaging protocols.

### 4.5 Cross‐Species Transfer and One Health Synergy

Many veterinary neuroimaging AI pipelines borrow model architectures and training strategies from human radiology through transfer learning, which can reduce data requirements and accelerate proof‐of‐concept development (Banzato et al. 2018; Lee et al. 2024). Translation can also be bidirectional: Naturally occurring disease in dogs, including gliomas and IVDD, can serve as a large‐animal context for evaluating algorithm robustness under real clinical variability (Huang et al. 2024). However, this bidirectional value is strongest when the target task, imaging endpoint and clinical outcome are explicitly defined for each species rather than assumed to be equivalent.

This bidirectional view operationalizes One Health in imaging AI by framing cross‐species work around shared validation goals, harmonized metadata and transparent governance rather than a one‐way transfer of human algorithms into veterinary practice (Ho 2022). Accordingly, cross‐species technology transfer should include domain adaptation and receiving‐species validation before performance claims are generalized.

### 4.6 Limitations, Methodological Requirements and Clinical Readiness

Despite encouraging reported performance metrics, most veterinary neuroimaging AI studies remain retrospective, single‐centre and based on small cohorts, which increases the risk of overfitting and limits generalizability (Akinsulie et al. 2024; Farhoodi et al. 2025). External test sets and prospective validation are uncommon, and reporting is often incomplete for clinically relevant aspects such as calibration, uncertainty and error trade‐offs under realistic disease prevalence. Differences in diagnostic equipment, pulse sequences, segmentation practices, breed morphology and case mix can also create dataset shift when models are moved across institutions (Sel et al. 2025).

Data sharing remains a practical barrier. Veterinary imaging data are often stored in institution‐specific systems, and clinically valuable annotations may exist in narrative reports rather than in standardized, machine‐readable formats. Thus, the central problem is not simply the absence of expert interpretation, but the difficulty of converting heterogeneous, siloed clinical records and imaging annotations into curated training datasets.

Addressing these limitations requires neuroimaging‐specific infrastructure rather than only better algorithms. Priority steps include multi‐centre collaboration, standardized acquisition and annotation practices, transparent reporting of validation design and harmonization frameworks analogous to the Brain Imaging Data Structure (BIDS) to improve reproducibility in veterinary neuroimaging (Zwanenburg et al. 2020; Gorgolewski et al. 2016). Until these requirements are met consistently, most imaging AI in veterinary neurology should be described as research‐stage or decision‐support candidate technology, not as a stand‐alone clinical diagnostic system.

## 5 AI in Electrophysiology and Seizure Prediction

**FIGURE 2:**
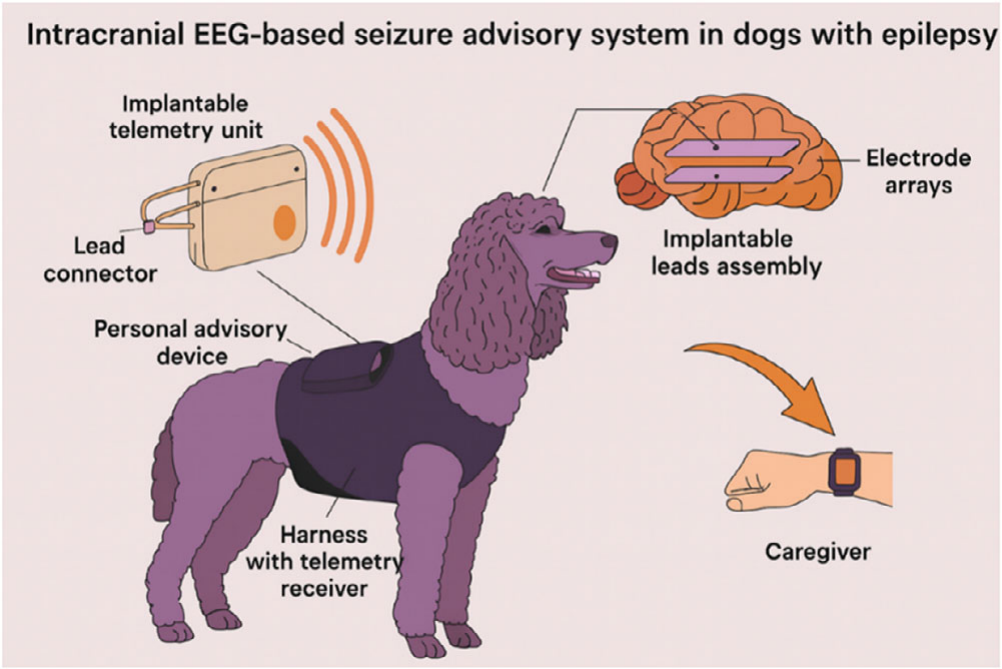
Conceptual schematic of an intracranial EEG‐based seizure advisory system (SAS) for canine epilepsy. Implanted subdural electrode arrays detect intracranial electrical activity and transmit the signals to an implantable telemetry unit, where iEEG data are processed to estimate seizure risk. When a seizure is forecast, the system wirelessly relays an alert to an external advisory device and then to the caregiver, enabling earlier intervention. This illustration is intended to show a possible workflow for continuous monitoring and remote alert delivery rather than a single commercially implemented system.

EEG and iEEG are important tools for diagnosis, monitoring and prognostication in epilepsy across human and veterinary neurology. These signals are high‐dimensional and non‐stationary time series, making them suitable for DL systems that can learn temporal and spectral features from complex electrophysiological data (Tan et al. 2025) (Figure 2). However, reported performance in this domain is highly sensitive to recording hardware, electrode placement, preprocessing choices, sedation status, artifact handling and cohort composition. Many veterinary studies therefore remain proof‐of‐concept rather than clinically deployable systems. Accordingly, interpretation should emphasize validation design, cross‐site generalizability, calibration and clinically meaningful error trade‐offs.

### 5.1 Automated EEG Interpretation

In human neurology, CNNs and recurrent neural networks (RNNs) have shown strong performance in interictal spike detection, seizure diagnosis and artifact rejection (Tjepkema‐Cloostermans et al. 2025). Veterinary applications follow the same general direction but are constrained by smaller datasets and more limited clinical integration. Algorithms have been developed to identify epileptiform discharges in canine scalp EEG, with performance approaching that of experienced electroencephalographers in selected settings, potentially reducing interpretation time and inter‐observer variability (Akinsulie et al. 2024). To strengthen clinical relevance, studies should report how performance changes under realistic conditions, including noise, sedation status, electrode montage, artifact burden and across‐institution acquisition differences, where feasible, external validation and calibration reporting are important because high accuracy alone may not translate into trustworthy decision support. In addition, temporal relevance mapping or related explainability visualizations should be considered in EEG applications because they can indicate which time windows, frequency bands or temporal–spectral features contributed most strongly to an algorithmic decision, thereby improving clinical interpretability and trust.

### 5.2 Seizure Forecasting

Seizure forecasting, defined as predicting ictal events minutes to hours before their occurrence, is a promising area in translational epilepsy research. In naturally occurring canine epilepsy, long‐term iEEG monitoring provides continuous real‐world seizure data and offers a valuable platform for developing predictive algorithms (Howbert et al. 2014). Nejedly et al. (2019) developed a subject‐specific CNN using both raw iEEG signals and spectrogram inputs. Their approach achieved mean sensitivities of approximately 79% while maintaining a low time‐in‐warning ratio, representing an improvement over random prediction (Nejedly et al. 2019). Other studies have compared CNN‐based approaches with RNNs, particularly long short‐term memory (LSTM) models, which can capture preictal temporal patterns and have achieved *F*‐scores above 0.7 in selected subjects (Mienye et al. 2024).

Collectively, these findings support the technical promise of DL for seizure prediction in both veterinary and human epilepsy. At the same time, forecasting metrics should be interpreted in relation to clinical utility because false alarms, time‐in‐warning burden and stability across recording days strongly influence whether a system is acceptable in practice. The acceptable error profile also depends on the intended setting. In hospital or ICU environments, a higher false‐alarm rate may be acceptable if sensitivity is prioritized to support preparation or pre‐emptive administration of rescue medication. In contrast, owner‐facing home monitoring systems require stricter control of false alarms because frequent alerts may increase anxiety, reduce adherence and undermine trust in the device. In addition, many forecasting pipelines are evaluated in small cohorts or within‐subject settings, so prospective evaluation and cross‐site testing remain necessary before routine veterinary translation.

### 5.3 Cross‐Species and Cross‐Modality Transfer

Canine epilepsy is a valuable comparative model for seizure prediction research because naturally occurring disease, long‐term monitoring and real‐world variability provide data streams that are difficult to reproduce in many human studies. DL models developed for human EEG can be adapted to canine data using domain adaptation methods, including Euclidean alignment or adversarial feature‐space matching (Wang et al. 2025). Incorporating canine iEEG into broader model training may improve robustness across heterogeneous datasets and reduce overfitting to specific recording conditions (Sumon et al. 2025). Nevertheless, cross‐species and cross‐centre deployment remains vulnerable to dataset shift caused by differences in skull geometry, electrode localization, amplifier characteristics, environmental noise and sensor placement. Therefore, mixed‐species training should not be interpreted as evidence of direct clinical transfer unless performance is revalidated in the receiving species and intended recording context.

### 5.4 Feature Learning and Interpretability

Although raw‐signal DL models are increasingly common, manually engineered features such as entropy measures, coherence and band‐limited power remain useful, especially when training datasets are limited (Zurdo‐Tabernero et al. 2023; Zhang et al. 2024). Hybrid approaches that combine deep feature extraction with engineered electrophysiological features have improved performance in some studies (Khansari et al. 2025; Daşdemir and Örnek 2025). Clinical acceptance, however, will depend not only on accuracy but also on interpretability. Techniques such as saliency mapping, layer‐wise relevance propagation and temporal relevance mapping can help identify the temporal or spectral patterns associated with predicted preictal states (Gurmessa and Jimma 2025; Sujatha Ravindran and Contreras‐Vidal 2023; Jemal et al. 2022). For clinically oriented deployment, interpretability should be paired with uncertainty estimates and calibration checks because an explainable but poorly calibrated model can still mislead decision‐making. Clear reporting of preprocessing, artifact handling, operating thresholds and model failure modes also improves reproducibility and cross‐study comparability.

### 5.5 Clinical Utility and Limitations

Seizure forecasting may support therapeutic management by enabling earlier intervention, adjustment of patient activity and improved timing of rescue medication or monitoring (Akras et al. 2025; Brinkmann et al. 2016). A veterinary‐facing application would be wearable or ambulatory monitoring, conceptually similar to Holter‐style monitoring, to identify patient‐specific seizure risk patterns in everyday environments. However, this remains an emerging research direction rather than a standard‐of‐care tool. Current barriers include limited veterinary datasets, individual heterogeneity in seizure dynamics, device‐related variability and insufficient prospective validation across clinical environments (Zurdo‐Tabernero et al. 2023; Zhang et al. 2024; Nasseri et al. 2020).

For this reason, veterinary electrophysiology AI should currently be framed as promising but early‐stage decision‐support technology. Translation will require larger multi‐centre datasets, standardized acquisition and annotation, prospective testing, calibration and uncertainty reporting and explicit definition of the intended user, whether veterinary professionals in a hospital setting or owners using home monitoring devices. This distinction is essential because the same sensitivity and false‐alarm profile may have very different clinical and emotional consequences depending on who receives and acts on the alert. Overall, seizure detection and forecasting remain among the clearest examples of bidirectional cross‐species AI innovation in neurology, but clinical deployment should proceed only after validation under realistic veterinary workflows.

## 6 AI for Movement Disorders, Gait Analysis and Pain Assessment

Quantitative evaluation of motor function and pain is important in veterinary neurology because conventional assessments often depend on clinician observation, owner reports and subjective scoring systems (E. V. Winkler et al. 2025). AI‐based methods can provide objective and high‐resolution measurements of locomotor patterns, posture, activity and pain‐related behaviour, supporting earlier detection and more consistent monitoring (Blättler et al. 2024; Reinstein et al. 2025). However, most veterinary applications remain research‐stage, and reported performance can decline when models are exposed to variation in recording conditions, patient morphology, species, breed, handler technique and clinical workflow. Therefore, proof‐of‐concept studies should be clearly distinguished from tools that are sufficiently validated for routine clinical decision support.

### 6.1 Wearable Sensor‐Based Gait Analysis

In veterinary neurology, inertial measurement units (IMUs) attached to collars, harnesses, limbs or the trunk have been combined with ML or DL models to classify gait abnormalities (Engelsman et al. 2022; X. Zhang et al. 2022). Recent studies suggest that CNNs trained on multi‐site IMU data can differentiate healthy gait patterns from those associated with neurological and orthopaedic disease, with multiclass classification accuracies above 90% in selected datasets (X. Zhang et al. 2022; Prasanth et al. 2021). Performance is influenced by sensor placement, device type, sampling rate, attachment tightness, sensor orientation drift and the activity being analysed, such as walking versus trotting. These findings are consistent with human wearable gait analytics in Parkinson's disease and stroke rehabilitation, where body‐mounted sensors can provide useful kinematic markers of disease severity and recovery (Prasanth et al. 2021; Salchow‐Hömmen et al. 2022; Ali et al. 2025).

Equine neurology and orthopaedics represent an especially important context for AI‐based gait and posture analysis. In horses, the ability to stand, move and maintain coordinated locomotion is central to welfare, athletic function and clinical decision‐making. Objective gait technologies are already more visible in equine practice than in many small‐animal settings, and sensor‐based or pressure‐based systems may provide decision‐support data for lameness localization, neurologic gait assessment and rehabilitation monitoring. In parallel, accelerometer‐ and gyroscope‐based datasets generated by smart collars and activity monitors may provide an underused resource for longitudinal detection of mobility changes in companion animals, although commercial data access, annotation quality and external validation remain major barriers.

### 6.2 Vision‐Based Gait and Posture Recognition

Computer vision approaches using 2D or 3D pose estimation have also been explored for movement analysis in veterinary patients (Han et al. 2025; Stenum et al. 2024; Lam et al. 2023). ML algorithms can detect subtle postural asymmetries by tracking joint trajectories, body segment angles, stride patterns and weight‐bearing behaviour over time (Han et al. 2025; Stenum et al. 2024). This aligns with human neurology research, where markerless motion capture is increasingly used to assess movement disorders and rehabilitation outcomes (Lam et al. 2023; Guo et al. 2022).

For veterinary translation, vision‐based systems must be validated across species and practice settings because camera placement, floor surface, lighting, coat length, body conformation, handler position and occlusion patterns can strongly influence pose estimation quality. These issues are particularly relevant for multi‐species deployment, where a model trained on one body shape or gait pattern may not generalize reliably to another. Standardized recording protocols and cross‐setting robustness testing are therefore essential before these systems can be used as clinical decision‐support tools.

### 6.3 Automated Pain Recognition

Pain recognition in veterinary medicine remains challenging, especially in species or contexts where behavioural signs are subtle, masked or unfamiliar to observers (Cascella et al. 2023). AI‐based facial and behavioural analysis has recently been explored as a way to support more consistent pain assessment (Cascella et al. 2023; Feighelstein et al. 2022; Gkikas and Tsiknakis 2023). Landmark‐based morphometric models, which quantify features such as ear position, orbital tightening, muzzle tension and facial geometry, have shown better performance than end‐to‐end CNN image classifiers in some feline acute pain datasets (Feighelstein et al. 2022; Martvel et al. 2024a; Steagall et al. 2023). Reported classification accuracies of approximately 77% for landmark‐based approaches compared with approximately 65% for CNN‐based classifiers suggest that anatomically informed feature extraction may be useful when training datasets are limited (Feighelstein et al. 2022; Martvel et al. 2024a). Explainable AI (XAI) studies have also indicated that the nose and mouth regions contribute substantially to model decisions, broadly aligning with components of the validated Feline Grimace Scale (Steagall et al. 2023; Martvel et al. 2024a).

Automated pain recognition is also highly relevant for laboratory species such as mice, rats and rabbits, in which subtle behavioural and facial indicators of pain may be less familiar to non‐specialist observers. Validated computer vision systems could support continuous welfare monitoring, improve consistency in humane endpoint assessment and reduce disagreement between research teams and veterinary staff by providing standardized and auditable observations. Comparable approaches have been explored in human neonatal and critical care settings, where facial expression analysis can assist pain assessment in non‐verbal patients when interpreted alongside clinical context (Gkikas and Tsiknakis 2023; Jiang et al. 2025; Lin et al. 2024). Nevertheless, pain models must be interpreted cautiously because facial features and behaviour may be confounded by sedation, stress, handling, species‐ or breed‐specific morphology and non‐pain illness states. For clinical credibility, studies should report how models handle these confounders and should include calibration or uncertainty reporting when outputs are used to support treatment decisions.

### 6.4 Translational Relevance

Gait, posture and pain analysis provide a useful foundation for cross‐species AI transfer because they involve measurable behavioural or biomechanical signals that can be compared across veterinary and human contexts (X. Zhang et al. 2022; Gkikas and Tsiknakis 2023). Algorithms designed for human gait abnormalities can be adapted for quadrupedal movement through species‐specific skeletal models, kinematic features and posture representations (Salchow‐Hömmen et al. 2022; Han et al. 2025; Guo et al. 2022). Conversely, veterinary locomotor datasets may expose algorithms to broader variation in body size, gait pattern, naturally occurring neurological disease and real‐world recovery trajectories than many controlled human rehabilitation datasets (Akinsulie et al. 2024; X. Zhang et al. 2022; Akbarein et al. 2025). This bidirectional value is strongest when endpoints are clearly harmonized, such as mobility, functional recovery, pain severity, welfare status or treatment response, rather than when performance is compared only through generic accuracy metrics.

### 6.5 Implementation Considerations

Despite encouraging performance in research settings, clinical adoption remains limited by hardware costs, workflow integration challenges, staff training requirements and the need for validation across diverse practice environments (Akinsulie et al. 2024; Farhoodi et al. 2025). Standardized protocols for sensor positioning, video recording, activity selection and annotation are needed to support multi‐centre data aggregation and model training (Gkikas and Tsiknakis 2023) (Table 2). Practical deployment also requires clear definition of who operates the system, how outputs are integrated into routine appointments and how clinicians should act when AI outputs conflict with clinical judgement. Without these implementation details, performance reported in controlled datasets may not translate into real‐world clinical benefit.

**TABLE 2:** Comparative AI applications in electrophysiology, gait and pain assessment: modalities, limitations and clinical maturity.

Application	Modality/signal	Species/setting	Main AI approach	Main finding	Main limitation	Clinical maturity	Ref
Automated EEG interpretation and seizure detection	Scalp EEG	Human EEG practice; canine EEG research	CNNs and RNNs for spike detection, seizure detection and artifact rejection	High performance in controlled settings; reduced interpretation burden	Performance depends on montage, noise, preprocessing and cross‐site consistency	Near‐clinical in human medicine; experimental in veterinary neurology	(Tan et al. 2025; Tjepkema‐Cloostermans et al. 2025; Akinsulie et al. 2024)
Seizure forecasting with iEEG	Long‐term iEEG	Naturally occurring canine epilepsy; human epilepsy cohorts	Subject‐specific CNNs; LSTM‐based RNNs	Mean sensitivity around 79%; *F*‐scores above 0.7 in selected settings	Small cohorts, false‐alarm burden, limited prospective and cross‐site validation	Experimental/translational research stage	(Nejedly et al. 2019; Howbert et al. 2014; Mienye et al. 2024; Akras et al. 2025; Brinkmann et al. 2016)
Cross‐species and interpretable EEG models	Scalp EEG and iEEG	Mixed human and canine datasets	Domain adaptation; hybrid deep plus handcrafted features; XAI	Mixed‐species training may improve robustness; interpretable features may support physiological plausibility	Dataset shift across species and sites; explainability does not replace validation	Early‐stage comparative research	(Wang et al. 2025; Sumon et al. 2025; Akras et al. 2025; Brinkmann et al. 2016)
Wearable sensor‐based gait analysis	IMU signals	Dogs with neurologic or orthopaedic disease; analogous human cohorts	CNNs on raw IMU series; ML on kinematic features	Over 90% accuracy reported in curated classification tasks	Sensitive to sensor placement, device type and recording variability	Promising for monitoring; not routine clinical standard	(Engelsman et al. 2022; X. Zhang et al. 2022; Prasanth et al. 2021; Salchow‐Hömmen et al. 2022; Ali et al. 2025)
Vision‐based gait and posture analysis	2D/3D video; pose estimation	Quadrupedal veterinary patients; human movement disorders	Markerless pose estimation with ML classifiers	Detects subtle gait and posture abnormalities not easily seen visually	Affected by camera setup, lighting, floor surface, coat type and occlusion	Research stage/early translational	(Han et al. 2025; Stenum et al. 2024; Lam et al. 2023)
Automated facial pain recognition	Facial images or video	Cats; neonatal and critical human settings	Landmark‐based morphometrics; CNN image classifiers	Landmark models outperform end‐to‐end CNNs in some feline datasets; clinically relevant facial regions identified	Confounding by stress, sedation, morphology and illness state; limited generalizability	Experimental decision‐support tool	(Cascella et al. 2023; Feighelstein et al. 2022; Gkikas and Tsiknakis 2023; Martvel et al. 2024b; Steagall et al. 2023)

## 7 AI in Prognostic Modelling and Clinical Decision Support

In neurology, accurate prediction is important for treatment planning, owner counselling, referral decisions and allocation of clinical resources (Giordano et al. 2021). AI can integrate clinical, imaging and electrophysiological data into predictive models that support risk stratification and decision‐making. Although these approaches are increasingly used in human neurology, veterinary applications remain less mature (Xiao et al. 2025). Most veterinary prognostic and decision‐support models are still proof‐of‐concept, and reported performance should be interpreted in relation to cohort size, case mix, disease prevalence, validation design and intended use. For clinical translation, it is essential to distinguish models that assist triage or risk stratification from tools that are sufficiently validated to influence high‐stakes decisions.

### 7.1 Epilepsy Etiology Classification

Determining whether a dog with newly developed seizures has idiopathic or structural epilepsy often requires advanced imaging, which may be costly or unavailable in many clinical settings (Charalambous et al. 2016). Recent studies suggest that Bayesian network models developed from clinical examination findings, seizure history and demographic data can achieve accuracies approaching 97% for distinguishing structural from idiopathic epilepsy (Flegel et al. 2024). Feature selection analyses identify age at seizure onset, seizure clustering and recent seizure activity as important predictors (Flegel et al. 2024; Jankauskas et al. 2025). These findings are conceptually similar to human neurology studies in which ML models such as random forests and gradient boosting have classified epilepsy etiology using accessible bedside data (Yousef et al. 2024; Fleischer et al. 2025).

However, high accuracy in retrospective datasets does not imply safe substitution for diagnostic imaging. Performance estimates may be inflated by spectrum bias, class imbalance and centre‐specific referral patterns, and many studies do not report calibration, predictive values or decision‐curve analyses that are directly relevant to clinical counselling. In veterinary practice, a false‐positive structural classification could contribute to unnecessary escalation or irreversible owner decisions, whereas a false‐negative result could delay imaging in a treatable case. Therefore, these models should currently be framed as triage‐support or risk‐stratification aids until they are externally validated across diverse clinical settings and reported with clinically interpretable error trade‐offs.

### 7.2 Surgical Prognosis

Forecasting functional recovery after spinal cord injury is important for case selection, referral decisions, treatment planning and owner counselling (Low et al. 2025; Shimizu et al. 2025). In dogs with severe thoracolumbar intervertebral disc extrusion (IVDE), an XGBoost model integrating neurological examination findings, MRI‐derived metrics and time from onset to surgery achieved an area under the curve (AUC) of 0.95 for predicting ambulatory recovery (Low et al. 2025). This approach resembles prognostic models in human spinal cord injury, where imaging and clinical data are combined to predict long‐term functional outcomes (Shimizu et al. 2025; Hossain et al. 2025).

Nevertheless, prognostic AUC values should be interpreted alongside external validation, calibration and robustness to variation in surgical timing, imaging protocols, surgeon experience, rehabilitation intensity and outcome definition. This is particularly important in cross‐species interpretation because ‘good recovery’ or ‘acceptable function’ may not carry the same clinical, welfare or quality‐of‐life meaning in animals and humans. Without these checks, models may overfit to local practice patterns rather than general biological prognosis. Prognostic outputs should therefore be communicated with uncertainty bounds and linked to actionable thresholds, such as identifying cases that may benefit from referral, additional imaging or intensified rehabilitation, rather than presented as deterministic predictions.

### 7.3 Multimodal Prognostic Integration

Integrating multimodal inputs such as clinical, imaging, electrophysiological and genetic data can improve predictive accuracy in some neurological contexts (Yousef et al. 2024; Guerra et al. 2025). In human multiple sclerosis, deep neural networks incorporating MRI lesion load, spinal cord atrophy and serum biomarkers have been used to predict disability progression more accurately than traditional grading systems (Yousef et al. 2024). Comparable integrated models in veterinary neurology are still largely theoretical but may become feasible as multi‐centre datasets expand (Akbarein et al. 2025; Xiao et al. 2025).

For veterinary translation, multimodal modelling will require careful handling of missing data, harmonization of acquisition protocols and explicit reporting of the incremental value provided by each modality. Otherwise, additional modalities may increase model complexity and overfitting risk without improving real‐world utility. Prospective designs and multi‐centre data sharing will be especially important for demonstrating generalizability across breeds, diagnostic equipment, imaging protocols and clinical settings.

### 7.4 Decision Support Systems

Beyond prognosis, AI may support case triage, diagnostic prioritization and treatment planning (Giordano et al. 2021; Golden et al. 2024). In veterinary neurology, the most realistic near‐term applications include prioritizing urgent imaging cases, assisting structured measurements, flagging cases for specialist review and supporting follow‐up planning. More ambitious applications, such as fully automated differential diagnosis or treatment recommendation systems, remain experimental. This distinction should be explicit because narrow, well‐defined decision‐support tools are easier to validate, monitor and govern than broad autonomous systems.

Implementation barriers include integration with PACS and practice‐management systems, model monitoring after deployment, clinician training and clear allocation of responsibility when AI suggestions conflict with clinical judgement. Accordingly, decision‐support systems should be designed for veterinarian‐in‐the‐loop use, where AI outputs inform but do not replace professional interpretation and clinical accountability.

### 7.5 Cautions and Clinical Responsibility

AI‐based prognostic models can improve consistency and support structured decision‐making, but they should augment rather than replace clinical judgement. Excessive reliance on models developed from biased, incomplete or poorly validated datasets may compromise patient care, particularly when outputs influence high‐stakes decisions such as surgery, long‐term treatment planning or euthanasia (Giordano et al. 2021; Xiao et al. 2025; Abbas et al. 2025; Bellamy 2023). For this reason, veterinary prognostic AI should be introduced as supervised decision support with clearly defined intended use, external and preferably prospective validation, calibration and uncertainty reporting and transparent communication of limitations to owners.

In high‐stakes contexts, evaluation should extend beyond discrimination metrics such as AUC or accuracy and include net clinical benefit, harm minimization and clinically meaningful error thresholds under realistic prevalence and cost constraints (Bellamy 2023). Until these requirements are met, prognostic AI in veterinary neurology should be considered a risk‐stratification aid rather than a stand‐alone basis for irreversible clinical decisions.

## 8 Educational and Laboratory Applications of AI

Although most AI applications in neurology focus on diagnosis, prognosis and monitoring, AI is also influencing veterinary education and laboratory research. In these settings, AI can support teaching, skill development, automated behavioural scoring and more standardized experimental assessment while aligning with ethical priorities such as the 3Rs principle of replacement, reduction and refinement (Akinsulie et al. 2024; González‐Gonzalo et al. 2022). However, educational and laboratory tools vary widely in maturity, and their value depends on reproducible data capture, standardized annotation, clear intended use and measurable outcomes such as learner performance, inter‐rater agreement, behavioural scoring reliability or animal welfare metrics.

### 8.1 Neuroanatomy and Neuropathology Teaching

Neuroanatomy is a challenging and conceptually demanding area of veterinary education, particularly when students are required to integrate normal structural anatomy with pathological and imaging findings. AI‐assisted 3D reconstruction methods derived from MRI datasets can generate interactive brain models that combine anatomical structures with clinically relevant imaging examples (Hadžiomerović et al. 2025). For example, DL segmentation of canine MRI scans can support the creation of high‐fidelity 3D models for educational use, helping students connect neuroanatomical structures with disease‐related imaging features. Comparable tools have been used in human medical education, where AI‐enhanced virtual and augmented learning platforms support neuroanatomy teaching and surgical simulation (Hadžiomerović et al. 2025; Choi et al. 2023).

These systems should be framed as teaching aids rather than replacements for expert instruction. Their effectiveness is best demonstrated through controlled educational outcomes, including improved learner performance, retention, diagnostic reasoning or inter‐rater agreement in anatomy identification tasks (Elendu et al. 2024). A further priority is the integration of AI literacy, validation principles and AI ethics into veterinary curricula and continuing professional education, so that future general practitioners and specialists can critically evaluate algorithmic tools rather than relying on marketing claims or unvalidated performance metrics.

### 8.2 Laboratory Animal Behaviour Analysis

In behavioural neuroscience, AI‐driven video and audio analysis can reduce reliance on labour‐intensive manual scoring and improve consistency in the detection and classification of animal behaviours (Isik and Unal 2023; Fazzari et al. 2025). In rat research, CNNs trained on ultrasonic vocalization (USV) spectrograms have been used to classify call types and link them to behavioural contexts, providing a more objective approach for assessing social and cognitive functions (Coffey et al. 2019; Fonseca et al. 2021). Automated systems can also identify subtle behavioural patterns that may be missed by human observers and may improve reproducibility across experiments when acquisition and annotation protocols are standardized (Hsieh et al. 2024; Voikar and Gaburro 2020).

By improving efficiency and reducing scoring variability, automation may support the 3Rs principle by helping researchers obtain reliable data with fewer animals or more refined endpoints (Verderio et al. 2023). Nevertheless, generalizability remains a key limitation. Behavioural models can be sensitive to camera placement, lighting, cage geometry, strain or breed differences, housing conditions and laboratory‐specific handling. Transparent reporting of acquisition conditions, annotation protocols, model validation and cross‐site testing is therefore necessary to avoid overstating the portability of performance (Okinda et al. 2020).

### 8.3 Welfare and Pain Monitoring in Research Settings

AI‐based pain and welfare recognition in laboratory and companion animal species is discussed in Section 6.3; therefore, this section is limited to its laboratory implementation context. In research settings, automated monitoring of mobility, posture, facial expressions and activity patterns can support more consistent welfare assessment and earlier detection of distress or functional decline (Feighelstein et al. 2022; Gill et al. 2025; Martvel et al. 2024b; Giordano et al. 2024). Because these systems may influence analgesia protocols or humane endpoint decisions, they should communicate uncertainty, minimize harmful error profiles and be validated against expert veterinary assessment before being used in routine animal welfare monitoring. This is especially important for laboratory species such as mice, rats and rabbits, where subtle signs of pain or distress may be less familiar to non‐specialist observers.

### 8.4 XAI in Education

In veterinary and human training contexts, XAI can serve both transparency and educational functions (Mastour et al. 2025; Li et al. 2025). For students, visualizations such as MRI heatmaps, saliency maps or EEG relevance maps can help illustrate which image regions or signal segments contributed to a model output (Mastour et al. 2025). XAI outputs may also help educators demonstrate feature–disease relationships using real clinical examples (Li et al. 2025, de Brito et al. 2025)

However, explainability methods can be misleading if they are treated as definitive causal evidence. For educational integrity, XAI visualizations should be presented as interpretive aids that require expert context, validation and human oversight. This framing is important because students should learn not only how AI systems generate outputs, but also how such outputs can fail, how validation should be judged and why clinician responsibility remains essential.

### 8.5 Cross Domain Knowledge Transfer

Many educational AI tools used in veterinary training originate from human medical education and are then adapted to veterinary curricula (Basran and Appleby 2024). Conversely, systems developed in veterinary contexts, especially those involving large‐animal gait analysis, behavioural phenotyping and laboratory animal monitoring, may inform human rehabilitation, orthopaedic training and translational neuroscience research (Hsieh et al. 2024; Min 2023). These applications support One Health goals when they create shared standards, reproducible annotation schemes and benchmarking datasets that allow methods trained in one domain to be tested transparently in another (Appleby and Basran 2022; Ho 2022; Verderio et al. 2023; Scott et al. 2023; Huang et al. 2024; Gill et al. 2025; Cross et al. 2024).

To make cross‐domain transfer more than a conceptual claim, future educational and laboratory AI studies should report intended use, validation setting, user group and measurable outcome improvements. Such reporting would help distinguish tools that genuinely improve learning, research reproducibility or welfare assessment from systems that are technically impressive but not yet educationally or scientifically validated.

## 9 Ethical, Methodological and Regulatory Considerations

AI in veterinary neurology may improve diagnosis, prognosis, monitoring and decision support, but its clinical use requires careful attention to ethics, methodological rigour and governance (Appleby and Basran 2022). Insights from human neurology can inform validation and safety practices, although veterinary medicine has distinct clinical, economic, regulatory and owner decision‐making contexts (Appleby and Basran 2022; Cohen and Gordon 2022). Ethical and regulatory risk increases as AI systems move from research‐stage prototypes towards tools that may influence clinical decisions. Therefore, safeguards should be proportional to intended use, clinical risk and the degree of autonomy assigned to the system.

### 9.1 Data Quality, Bias and Representativeness

The reliability of any AI system depends strongly on the quality, diversity and representativeness of its training data. Bias may arise when datasets over‐represent specific breeds, species, disease severities, referral populations or imaging protocols, reducing performance in underrepresented groups (Norori et al. 2021). For example, a model trained predominantly on brachycephalic dog breeds may perform less reliably in dolichocephalic breeds because of breed‐associated anatomical differences. In veterinary neurology, small sample sizes further increase the risk of overfitting, where a model learns dataset‐specific artifacts rather than clinically generalizable disease patterns (Norori et al. 2021; Cross et al. 2024).

Addressing these limitations requires multi‐centre collaboration, standardized data collection and annotation and intentional inclusion of diverse patient populations (Appleby and Basran 2022; Norori et al. 2021). In addition to cohort size, veterinary datasets can show substantial distribution shift driven by breed morphology, heterogeneous acquisition protocols, variable diagnostic equipment and inconsistent clinical labelling practices. For this reason, reporting should extend beyond headline accuracy and include dataset composition, breed and age distribution, disease severity, missingness patterns, preprocessing steps and validation context, so readers can judge where the model is likely to fail (Maciulevičius et al. 2025).

### 9.2 Transparency and Explainability

Black‐box AI models may achieve high predictive performance but lack interpretability, which is problematic in neurology when outputs may influence treatment decisions, surgery, long‐term management or euthanasia (Cohen and Gordon 2022; Farahani et al. 2022; Lee and Lee 2024). XAI techniques, including saliency mapping in neuroimaging and temporal relevance mapping in EEG, can help identify the image regions, signal segments or temporal–spectral features that contributed to model predictions (Farahani et al. 2022; A and R 2023). In veterinary practice, this transparency can support clinician trust and improve communication with owners.

However, explainability should not be presented as proof of causality or model safety. Saliency‐style outputs can be unstable across architectures, preprocessing choices and input perturbations. Therefore, XAI should be interpreted as supportive evidence and combined with rigorous validation, uncertainty reporting and clinically meaningful error analysis. Practical transparency also requires clear documentation of intended use, target population, input requirements, operating thresholds, known failure modes and the role of the veterinarian in interpreting AI outputs (Ray 2026).

### 9.3 Ethical Implications of Prognostic AI

Prognostic AI requires particular caution in veterinary neurology because predicted outcomes may influence irreversible decisions, including surgical intervention, intensive treatment, long‐term care or euthanasia (Cohen and Gordon 2022; Coghlan and Quinn 2024). Excessive reliance on inadequately validated systems may lead to premature or inappropriate decisions, especially if outputs are perceived as objective or definitive. Ethical implementation therefore requires independent validation, transparent communication of uncertainty and integration of AI outputs into a broader clinical decision‐making process.

Prognostic models should be framed as decision‐support tools rather than decision‐making systems. They should disclose expected trade‐offs between false positives and false negatives under realistic disease prevalence and should report calibration because owners and clinicians may use predicted probabilities to guide high‐stakes choices. Poorly calibrated models can be ethically problematic even when discrimination metrics such as AUC appear strong (González‐Gonzalo et al. 2022; Moreno‐Sánchez et al. 2026). In practice, model outputs should be communicated as probabilistic estimates that require clinical interpretation rather than deterministic conclusions.

### 9.4 Validation and Regulatory Pathways

In human neurology, agencies such as the US Food and Drug Administration (FDA) and the European Medicines Agency (EMA) require performance validation, risk assessment and post‐market surveillance for many AI‐enabled medical devices (Zhang et al. 2025). Veterinary medicine currently lacks comparable neurology‐specific regulatory pathways, and many AI tools fall under broader software, medical device or professional responsibility frameworks rather than dedicated veterinary AI regulation (Appleby and Basran 2022). For clinically oriented deployment, evaluation should include external validation across institutions, prospective or temporally separated testing where feasible, calibration and uncertainty reporting and robustness checks for distribution shift across breeds, diagnostic equipment, acquisition protocols and clinical settings.

However, veterinary AI governance is likely to develop less through centralized regulation alone and more through a combination of professional guidance, institutional policies, market forces, developer responsibility and individual clinician accountability. Specialty colleges and professional organizations can issue position statements, consensus recommendations and reporting standards, but their ability to enforce legally binding regulation may be limited. This reality supports a staged approach in which developers begin with narrow, well‐defined applications that are defensible from validation and monitoring perspectives, rather than broad autonomous systems with poorly specified clinical boundaries. Even before formal regulation matures, adopting a human‐style safety mindset, minimum reporting standards, veterinarian‐in‐the‐loop workflows and post‐deployment performance monitoring can reduce avoidable harms.

### 9.5 Data Privacy and Ownership

Veterinary neurology records may contain owner information, clinic identifiers, imaging metadata and other data that can be linked to individuals or institutions (Sobkowich 2025). Although privacy standards in veterinary medicine are generally less formalized than those applied to human medical data, such as HIPAA or GDPR, ethical data management remains important. This includes informed consent where appropriate, secure storage, controlled access, de‐identification and explicit data‐sharing agreements for multi‐institutional collaborations (Sobkowich 2025; Nieuwland and Meijboom 2019).

Privacy and ownership issues can also limit multi‐centre model development. Federated learning and secure data environments may support collaboration while reducing raw‐data transfer, but they still require governance frameworks, audit trails, quality control and clarity regarding data ownership and permissible downstream use (Kumar et al. 2025). These safeguards are especially important when veterinary datasets are used beyond their original clinical purpose, including algorithm development, commercial deployment or cross‐species translational research.

### 9.6 Cross‐Species Research Ethics

When veterinary patients are used as translational models for human disease, AI research should ensure reciprocal benefit and avoid treating animal data as merely convenient substitutes for human datasets (Nieuwland and Meijboom 2019; A. S. Winkler et al. 2025). Cross‐species projects should state the expected direction of benefit, whether human‐to‐veterinary, veterinary‐to‐human or bidirectional, and should define safeguards for fair credit, data stewardship and responsible interpretation of results (Milazzo et al. 2025).

Ethically aligned One Health AI should improve or plausibly support healthcare for both animals and humans. When veterinary datasets are used to stress‐test or refine human algorithms, study design should also justify the veterinary clinical value of the work, such as improved diagnostic tools, monitoring systems, prognostic support or treatment planning in veterinary neurology (Xiao et al. 2025). This approach helps ensure that cross‐species AI development remains collaborative, clinically meaningful and ethically defensible.

## 10 Discussion

AI is beginning to influence diagnostic, prognostic, monitoring and educational aspects of veterinary neurology in ways that parallel, but do not fully mirror, its development in human neurology (Bösel et al. 2025; Benjamens et al. 2020). The available evidence highlights a reciprocal translational relationship: Veterinary medicine can adapt human‐developed AI frameworks, while human neurology may benefit from naturally occurring animal disease models that provide longitudinal, clinically realistic data and pathological diversity that are difficult to capture in controlled experimental settings (Löscher 2022). However, this transfer is not automatic. Differences in anatomy, acquisition protocols, clinical workflows, owner decision‐making and species‐specific outcome definitions mean that cross‐domain transfer requires explicit adaptation, careful validation and transparent reporting before claims of clinical readiness can be justified.

### 10.1 Comparative State of Development

In human neurology, selected AI systems have reached clinical deployment and regulatory authorization, particularly in imaging‐based detection, triage and segmentation workflows, with additional development in electrophysiology and prognostic modelling (Bösel et al. 2025; Benjamens et al. 2020). These systems are generally supported by larger datasets, more standardized pipelines and more mature validation norms than are currently available in veterinary neurology. In contrast, veterinary neurology remains at an earlier stage of adoption (Tveit et al. 2023; Onciul et al. 2025). Most published studies are retrospective, single‐centre and based on relatively small cohorts, which limits generalizability and increases the risk of overfitting (Akinsulie et al. 2024; Farhoodi et al. 2025).

Even when reported accuracy appears high, limited external validation, incomplete calibration reporting and scarce prospective testing reduce confidence in real‐world performance (Gulzar and Hussain 2023; Basran and Appleby 2024). Nonetheless, proof‐of‐concept studies in brain tumour classification, spinal lesion grading, seizure prediction, gait analysis, pain recognition and prognostic modelling show technical feasibility and justify continued development (Blättler et al. 2024). The key conclusion is therefore not that veterinary neurology AI is ready for autonomous clinical use, but that several narrowly defined applications may be suitable for staged development as supervised decision‐support tools.

### 10.2 Translational Synergy and One Health

Cross‐species technology transfer is a central theme of this review. Canine epilepsy provides a valuable setting for evaluating seizure detection and forecasting algorithms under long‐term, real‐world conditions that are difficult to reproduce in many human trials (Brinkmann et al. 2016). In parallel, transfer learning from human neuroimaging datasets may accelerate veterinary diagnostic AI by reducing dependence on large, fully annotated veterinary cohorts (Banzato et al. 2018; Lee et al. 2024). This reciprocity aligns with One Health principles, which emphasize coordinated knowledge exchange across human and veterinary medicine (Pereira et al. 2023).

However, One Health synergy is most convincing when it is operationalized through shared data standards, harmonized metadata, transparent validation and clearly defined endpoints (Ho 2022). Cross‐species transfer should not assume that similar imaging findings, electrophysiological patterns or functional outcomes have identical clinical meaning across humans, dogs, cats, horses or laboratory species. For example, spinal imaging abnormalities, seizure‐warning thresholds, gait impairment and pain behaviours may carry different welfare, functional and decision‐making implications across species (Low et al. 2025; Feighelstein et al. 2022; Martvel et al. 2024b). Therefore, future comparative AI studies should define whether the intended value is human‐to‐veterinary transfer, veterinary‐to‐human transfer or bidirectional validation, and should report how performance is tested in the receiving species and clinical context

### 10.3 Clinical Translation and Implementation Pathways

The clinical implications of veterinary neurology AI are best understood as part of the discussion rather than as a separate post‐discussion section. The most defensible near‐term role for AI is supervised decision support, with clearly defined intended use, validated performance boundaries and transparent communication of uncertainty (Appleby and Basran 2022; Basran and Appleby 2024; Cohen and Gordon 2022). In neuroimaging, AI may assist radiologists and neurologists by standardizing measurements, prioritizing time‐sensitive cases or acting as a structured second reader (Monsour et al. 2022; Herr et al. 2024; Pérez‐Sanpablo et al. 2025; Nourizadeh et al. 2025). In epilepsy, seizure detection or forecasting systems may eventually support rescue‐medication planning or risk monitoring, but their acceptable false‐alarm profile will differ between ICU or hospital use and owner‐facing home monitoring (Howbert et al. 2014; Kerr et al. 2024; Löscher 2022). In gait and pain assessment, wearable sensors, markerless video analysis and automated facial or behavioural scoring may provide objective follow‐up metrics, provided that recording conditions and endpoints are standardized (Zhang et al. 2022; Prasanth et al. 2021).

Prognostic tools also have potential value for owner counselling, referral decisions and treatment planning, but they require particular caution (Flegel et al. 2024; Low et al. 2025). Predictions should be presented as calibrated probabilities or uncertainty‐bounded estimates rather than deterministic conclusions (Khosravi et al. 2024, González‐Gonzalo et al. 2022, Moreno‐Sánchez et al. 2026). This is especially important in veterinary neurology because AI outputs may influence emotionally and ethically charged decisions, including surgery, intensive treatment, long‐term care or euthanasia (Cohen and Gordon 2022; Coghlan and Quinn 2024). Therefore, AI should support clinician–owner communication rather than replace professional judgement or shared decision‐making.

### 10.4 Methodological and Ethical Priorities

In cross‐domains, the same methodological pattern emerges: Reported performance is encouraging, but clinical readiness remains limited by small datasets, internal validation, incomplete calibration and weak evidence of generalizability (Gulzar and Hussain 2023; Basran and Appleby 2024; Akinsulie et al. 2024; Farhoodi et al. 2025). These limitations have already been discussed throughout the manuscript, so the central priority is consolidation rather than repetition. Future work should emphasize external multi‐centre validation, prospective or temporally separated testing, calibration and uncertainty reporting, performance stratification by breed and disease severity and clinically interpretable error analysis (Norori et al. 2021; Cross et al. 2024; Maciulevičius et al. 2025).

Explainability can support trust, education and owner communication, but it cannot substitute for validation. Saliency maps, EEG relevance maps or imaging heatmaps should be interpreted as supportive tools that require clinical context (Mastour et al. 2025; Farahani et al. 2022; A and R 2023). Similarly, veterinarian‐in‐the‐loop workflows should be treated as a core safety requirement, not an optional implementation feature. This is particularly important when AI systems are used in high‐stakes diagnostic or prognostic settings (Appleby and Basran 2022; Basran and Appleby 2024; Cohen and Gordon 2022; Ray 2026).

### 10.5 Addressing the Implementation Gap

For AI in veterinary neurology to move from isolated demonstrations to trustworthy clinical support, several practical steps are needed (Akbarein et al. 2025). First, developers should begin with narrow, well‐defined applications that are easier to validate, monitor and integrate into clinical workflows, rather than broad autonomous systems with poorly specified clinical boundaries (Hill 2024; Bajaj et al. 2023). Second, standardized protocols for imaging, electrophysiology, wearable sensing, video acquisition, annotation and outcome reporting are essential for reproducible multi‐institutional development (Akbarein et al. 2025; Bösel et al. 2025). Third, privacy‐preserving collaboration, including federated learning where appropriate, may help overcome data fragmentation while reducing the need for raw‐data sharing (Kumar et al. 2025). Fourth, AI systems should be integrated into existing workflows with minimal disruption, clear documentation, decision logging, post‐deployment monitoring and defined responsibility when AI outputs conflict with clinical judgement (Akinsulie et al. 2024; Akbarein et al. 2025).

A final implementation priority is education. Veterinary curricula and continuing professional development should include AI literacy, validation principles, limitations of algorithmic tools and AI ethics so that clinicians can critically evaluate emerging technologies rather than relying on marketing claims or headline accuracy (Elendu et al. 2024; Mastour et al. 2025; Li et al. 2025). Together, these steps would help shift veterinary neurology AI from promising research prototypes towards transparent, validated and clinically accountable decision‐support systems within a One Health framework (Ho 2022; Verderio et al. 2023).

## 11 Conclusion and Future Directions

AI is emerging as a promising decision‐support approach in veterinary neurology, with potential applications in diagnostic imaging, electrophysiology, seizure forecasting, gait analysis, pain assessment, morphometric biomarker discovery and prognostic modelling. Across these domains, available studies show that AI can detect complex patterns in imaging, signal, behavioural and clinical data, particularly in narrowly defined tasks. However, the current evidence should be interpreted as demonstrating technical feasibility rather than routine clinical readiness, because most veterinary applications remain proof‐of‐concept and are supported by limited validation.

The comparative perspective highlights a bidirectional exchange of value between human and veterinary neurology. Human medicine contributes established algorithms, larger annotated datasets and more mature validation and regulatory experience, which may accelerate veterinary AI development through transfer learning and domain adaptation. In return, veterinary neurology provides naturally occurring disease models, including canine epilepsy, spinal cord injury, CM, gait disorders and pain‐related conditions, which may offer clinically realistic settings for longitudinal monitoring and algorithm robustness testing. This cross‐species value is most defensible when models are validated in the receiving species, endpoints are species‐appropriate and benefits for veterinary patients are clearly defined rather than assumed.

Despite encouraging findings, wider clinical adoption remains limited by small and heterogeneous datasets, inconsistent acquisition and annotation practices, limited external and prospective validation, incomplete calibration and uncertainty reporting and underdeveloped veterinary‐specific governance pathways. Future progress should therefore prioritize multi‐centre collaboration, standardized data and reporting practices, privacy‐preserving approaches such as federated learning, prospective validation, XAI and post‐deployment monitoring for dataset shift and model drift. AI should be positioned as veterinarian‐in‐the‐loop decision support, not as an autonomous replacement for clinical judgement, especially when outputs may influence high‐stakes decisions such as surgery, long‐term treatment planning or euthanasia. With stronger methodological standards, ethical safeguards, AI literacy training and collaborative One Health frameworks, veterinary neurology AI can progress from experimental demonstrations towards transparent, validated and clinically accountable support tools that may also inform advances in human neurological medicine.

## Author Contributions

**Kianoush Saberi**: conceptualization, writing – original draft, and writing – review and editing. **Rosull Saadoon Abbood**: writing – original draft and writing – review and editing. **Sarah F. Al‐Taie**: writing – original draft and writing – review and editing. **Aseel Smerat**: writing – original draft and writing – review and editing. **Abdullaev Makhmudjon Mukhamedovich**: writing – original draft and writing – review and editing. **Maksudova Malika Khamdamjonovna**: writing – original draft and writing – review and editing. **Mohammad Hedayatinia**: writing – original draft and writing – review and editing. **Arash Zarei**: writing – original draft and writing – review and editing. **Mehrdad Nourizadeh**: writing – original draft and writing – review and editing. **Amir Arsalan Ghahari**: supervision and writing – review and editing. **Mehrdad Neshat Gharamaleki**: supervision and writing – review and editing. **Fatemeh Malekinejad**: writing – original draft and writing – review and editing. **Reza Akhavan‐Sigari**: writing – original draft and writing – review and editing.

## Funding

The authors have nothing to report.

## Ethics Statement

The authors have nothing to report.

## Consent

The authors have nothing to report.

## Conflicts of Interest

The authors declare no conflicts of interest.

## Data Availability

Data sharing is not applicable to this article as no datasets were generated or analysed during the current study.
